# Neighborhood disadvantage and chronic disease management

**DOI:** 10.1111/1475-6773.13092

**Published:** 2018-11-23

**Authors:** Shayla N. M. Durfey, Amy J. H. Kind, William R. Buckingham, Eva H. DuGoff, Amal N. Trivedi

**Affiliations:** ^1^ The Warren Alpert Medical School Brown University Providence Rhode Island; ^2^ School of Medicine and Public Health University of Wisconsin Madison Wisconsin; ^3^ School of Public Health University of Maryland College Park Maryland; ^4^ Brown School of Public Health Providence Rhode Island

**Keywords:** social determinants of health, geographic/spatial factors/small area variations, Medicare

## Abstract

**Objective:**

To assess the relationship between a composite measure of neighborhood disadvantage, the Area Deprivation Index (ADI), and control of blood pressure, diabetes, and cholesterol in the Medicare Advantage (MA) population.

**Data Sources:**

Secondary analysis of 2013 Medicare Healthcare Effectiveness Data and Information Set, Medicare enrollment data, and a neighborhood disadvantage indicator.

**Study Design:**

We tested the association of neighborhood disadvantage with intermediate health outcomes. Generalized estimating equations were used to adjust for geographic and individual factors including region, sex, race/ethnicity, dual eligibility, disability, and rurality.

**Data Collection:**

Data were linked by ZIP+4, representing compact geographic areas that can be linked to Census block groups.

**Principal Findings:**

Compared with enrollees residing in the least disadvantaged neighborhoods, enrollees in the most disadvantaged neighborhoods were 5 percentage points (*P *< 0.05) less likely to have controlled blood pressure, 6.9 percentage points (*P* < 0.05) less likely to have controlled diabetes, and 9.9 percentage points (*P* < 0.05) less likely to have controlled cholesterol. Adjustment attenuated this relationship, but the association remained.

**Conclusions:**

The ADI is a strong, independent predictor of diabetes and cholesterol control, a moderate predictor of blood pressure control, and could be used to track neighborhood‐level disparities and to target disparities‐focused interventions in the MA population.

## 1 INTRODUCTION

Persons living in disadvantaged neighborhoods have increased risk of worse health outcomes, including uncontrolled blood pressure, blood sugar, and cholesterol.^1^, ^2^ Most health insurers in the United States, including all Medicare Advantage (MA) plans, routinely report on rates of hypertension, diabetes, and cholesterol control as important measures of the quality of care. Understanding the association between neighborhood disadvantage and quality measures is important for MA plans, which receive bonuses and penalties based on achievement of these measures.^3^ This evidence may encourage MA plans to target quality improvement and population health interventions to persons living in disadvantaged neighborhoods. Medicare Advantage plans, which now enroll about one‐third of the Medicare population and have expanded fastest in disadvantaged areas,^4^ are uniquely suited to address disparities in outcomes. In contrast to the fee‐for‐service (FFS) Medicare program, MA plans receive capitated payments to provide comprehensive, managed care at a lower out of pocket cost to enrollees.^4^ Medicare Advantage disproportionately enrolls racial/ethnic minorities and those with low socioeconomic status (SES).^5^ However, the extent to which neighborhood disadvantage matters for the MA population is not known.

The relationship between neighborhood context and health is complex and mediated by factors including safety, walkability, stress, and access to health care, nutritious foods, and recreation.^6^ This relationship is further complicated by interaction with individual socioeconomic factors, including race/ethnicity. It is theorized that segregation of disadvantaged groups into resource‐poor areas with substandard housing, barriers to nutritious food, and unsafe outdoor spaces perpetuates structural inequalities. These neighborhoods often become cut off from key opportunities including mainstream employment and community social support.^7^, ^8^ The lack of these fundamental factors has the potential to induce stress and worsen disease outcomes.^8^ Neighborhood disadvantage is a metric that reflects neighborhood‐level social determinants of health, and has been linked to poor health outcomes and early mortality.^9^, ^10^, ^11^, ^12^, ^13^

Although studies show that neighborhood disadvantage impacts health even after adjustment for individual SES,^11^, ^14^ the association between area‐level and individual SES is bidirectional and the separate contribution of each component is not always clear.^15^ Quantifying the effect of neighborhood disadvantage is further challenged by the lack of an accepted, accessible indicator. Although patient‐level measures of neighborhood disadvantage are often unavailable in health data, some health agencies outside the United States have developed and validated geographic indices of neighborhood disadvantage to identify socioeconomic disparities and target quality improvement efforts.^16^ The Area Deprivation Index (ADI) is a validated indicator of neighborhood disadvantage that may be used similarly in the United States.^10^, ^17^ A composite index created from individual socioeconomic factors not only better captures the multidimensional construct of neighborhood disadvantage, but has greater validity and explanatory power than single individual measures.^17^ The ADI is also widely accessible to front‐line providers, researchers, and policy makers through the Neighborhood Atlas, and benefits from ease of use.^18^

We evaluated the relationship between a publicly available composite measure of neighborhood disadvantage, the ADI, and control of blood pressure, diabetes, and cholesterol in the MA population. We further assessed whether adjustment for individual SES attenuated this relationship, and whether the relationship between neighborhood disadvantage and health outcomes differed across racial and ethnic groups.

## 2 METHODS

### 2.1 Data sources and study population

We obtained person‐level Medicare Healthcare Effectiveness Data and Information Set from the Center for Medicare & Medicaid Services (CMS) for 2013. These data included 623 363 MA plan enrollees in 522 contracts who were eligible for one or more dichotomous measures of blood pressure control, diabetes control, and/or cholesterol control. Although CMS measures quality indicators at the contract level (including one or more plans), we used the more widely understood term “plan” to denote “contract” in this manuscript. We matched these data to a measure of neighborhood disadvantage known as the ADI using enrollees’ nine‐digit ZIP Code of residence.^10^ This match yielded an ADI value for 85 percent of the sample, but we retained individuals with a missing ADI value for analyses (see “Statistical Analysis” below). We used nine‐digit ZIP Codes because compared to five‐digit ZIP Codes, they represent more compact areas and are more accurate proxies for single neighborhoods. The ADI is a publicly available composite score generated from 17 socioeconomic variables collected in the 2013 American Community Survey and available through the University of Wisconsin.^10^, ^17^ See Appendix S1 for a list of variables included in the ADI. Poverty, income, and education were the largest contributors to the index.^17^

Enrollee demographic information including age, sex, race/ethnicity, nine‐digit ZIP Code of residence, dual eligibility, and disability were obtained from the Medicare Beneficiary Summary File. The data were then matched to the 2013 NCHS Urban‐Rural Classification Scheme for Counties by Federal Information Processing Standards code.^19^ The Urban‐Rural Classification Scheme is a measure of rurality, based on county population, developed for the evaluation of health differences across urban and rural areas.^19^

The final dataset included 175 229 enrollees in 457 plans eligible for blood pressure control, 269 789 enrollees in 453 plans eligible for diabetes control, and 196 765 enrollees in 379 plans eligible for cholesterol control. See Appendix S2 for more details on the construction of the study population.

### 2.2 Study variables

Dependent variables included dichotomous measures of blood pressure <140/90 mmHg for enrollees with hypertension, hemoglobin A1c <9.0 percent for enrollees with diabetes, and low‐density lipoprotein cholesterol <100 mg/dL for enrollees with a prior‐year history of a cardiac event.

The primary independent variable was the ADI. Because the ADI is a relative measure, neighborhood disadvantage was split into 20 groups of equal sample sizes, as is standard in ADI applications.^10^ The approximately 15 percent of the study population who could not be linked with an ADI value was coded with an indicator variable for missing. Additional factors included sex, race/ethnicity (black, white, Hispanic, Asian/Pacific Islander, American Indian/Alaska Native, other, and unknown), dual eligibility, disability, geographic region of residence, and rurality. Race/ethnicity was measured using the Research Triangle Institute Race Code, which uses surname analysis to improve the identification of Asian and Hispanic enrollees.^20^ Enrollees were identified as dual eligible if they were enrolled in Medicaid or any cost sharing program in at least 1 month during the study year. Enrollees were defined as disabled if they were originally enrolled in Medicare for disability and/or end stage renal disease rather than for age >65 years. Rurality was assessed using a six category measure ranging from most urban (large central metropolitan) to most rural (noncore micropolitan) by county population.^19^

### 2.3 Statistical analysis

To understand the composition of the least and most disadvantaged neighborhoods, we analyzed the difference between the characteristics of enrollees in the least and most disadvantaged quintiles of neighborhood disadvantage using chi‐square tests. We identified significant predictors of blood pressure, diabetes, and cholesterol control at the *P* < 0.05 level using bivariate logistic regression (Table 2). We then tested this association, adjusting for other individual and area‐level factors in generalized linear models that specified a binomial distribution for the outcomes and an identity link function to present estimates on the risk‐difference scale. The models used generalized estimating equations to adjust the standard errors for clustering at the county level. We conducted a sensitivity analysis using multiple imputation to analyze differences between adjusted models ran with missing ADI scores compared to imputed ADI scores (Appendix S2: Table S2). ADI (in ventiles) was the imputed variable in the models, and twenty imputations were created. We included an interaction term between ADI and race/ethnicity to determine whether the association between ADI and outcomes varied by race/ethnicity. Lastly, we analyzed interaction terms between ADI and dual eligibility as well as ADI and disability to determine whether area‐level neighborhood disadvantage overlaps with individual social risk.

All analyses were conducted using *STATA*, Version 14.2 (StataCorp. 2015. *Stata Statistical Software: Release 14*. College Station, TX: StataCorp LP.) and *SAS*® software, Version 9.4 of the SAS System for Windows (Copyright © 2013 SAS Institute Inc., Cary, NC, USA). The study protocol was approved by Brown University's IRB.

## 3 RESULTS

The characteristics of enrollees in the most and least disadvantaged neighborhoods are described in Table 1. Within the overall study cohort, 13.3 percent of enrollees were black, 48.7 percent were female, 22.5 percent were dual eligible, and 30.8 percent were disabled. Compared to the group of enrollees with a linked ADI score, those missing an ADI score included higher proportions of white and rural enrollees. The groups otherwise shared similar characteristics (Appendix S2: Table S1). Compared to the least disadvantaged neighborhoods, the most disadvantaged neighborhoods consisted of higher proportions of black enrollees in the blood pressure (34 vs 9 percent *P* < 0.001), diabetes (28 vs 8 percent; *P* < 0.001), and cholesterol (21 vs 5 percent; *P* < 0.001) cohorts (Table 1). Similarly, the most disadvantaged neighborhoods had higher proportions of dual eligible enrollees in the blood pressure (44 vs 24 percent, *P* < 0.001), diabetes (41 vs 14 percent, *P* < 0.001), and cholesterol (35 vs 10 percent, *P* < 0.001) cohorts, as well as higher proportions of disabled enrollees in the blood pressure (38 vs 16 percent; *P* < 0.001), diabetes (48 vs 20 percent; *P* < 0.001), and cholesterol (47 vs 19 percent; *P* < 0.001) cohorts (Table 1). A higher proportion of enrollees in the most disadvantaged neighborhoods resided in the most rural areas in the blood pressure (6.0 vs 0.0 percent *P* < 0.001), diabetes (5.0 vs 0.0 percent; *P* < 0.001), and cholesterol cohorts (5.0 vs 0.0 percent; *P* < 0.001) (Table 1).

**Table 1:** Socioeconomic and demographic characteristics of Medicare Advantage enrollees eligible for control measures in the least and most disadvantaged neighborhoods^a^

	Blood pressure control			Diabetes control			Cholesterol control		
	Overall (n = 182 012)	Least disadvantaged quintile (n = 30 489)	Most disadvantaged quintile (n = 30 491)	Overall (n = 272 033)	Least disadvantaged quintile (n = 46 213)	Most disadvantaged quintile (n = 46 227)	Overall (n = 200 356)	Least disadvantaged quintile (n = 33 926)	Most disadvantaged quintile (n = 33 930)
Sex									
Female	57	54	60	51	47	56	37	31	44
Race/Ethnicity									
Non‐Hispanic white	67	66	45	57	52	48	70	68	57
Black/African American	15	9	34	15	8	28	10	5	21
Hispanic	12	11	18	19	18	20	13	10	19
Asian/Pacific Islander	4	12	2	6	17	2	4	13	2
American Indian/Alaska Native	0	0	0	0	0	0	0	0	0
Other	1	2	1	2	4	1	1	3	1
Unknown	0	1	0	1	1	0	0	1	0
Dual eligibility									
Dual eligible	27	22	44	24	14	41	19	10	35
Original reason for medicare enrollment									
Disability and/or ESRD	26	16	38	34	20	48	33	19	47
Rurality									
Most urban (large central metro)	31	42	39	45	66	40	37	56	37
Large fringe metro	18	30	10	18	16	13	21	25	14
Medium metro	26	22	27	21	15	27	24	15	27
Small metro	11	4	10	7	2	9	8	3	9
Micropolitan	8	2	8	6	1	8	6	1	9
Most rural	5	0	6	3	0	5	4	0	5
Geographic region									
Northeast	19	32	14	13	13	13	14	15	13
Midwest	21	10	23	14	2	21	15	3	20
South	34	18	47	23	6	41	30	8	49
West	27	40	16	49	79	25	40	74	19

a All values are percentages and may not add to 100 due to rounding. Neighborhood deprivation is derived from the Area Deprivation Index score, which was split into five equally sized quintiles. The least (1st) and most (5th) disadvantaged quintiles are presented here. The 2nd through 4th quintiles are not presented. All least disadvantaged/most disadvantaged comparisons are significant at a level of *P* < 0.001.

**Figure 1:**
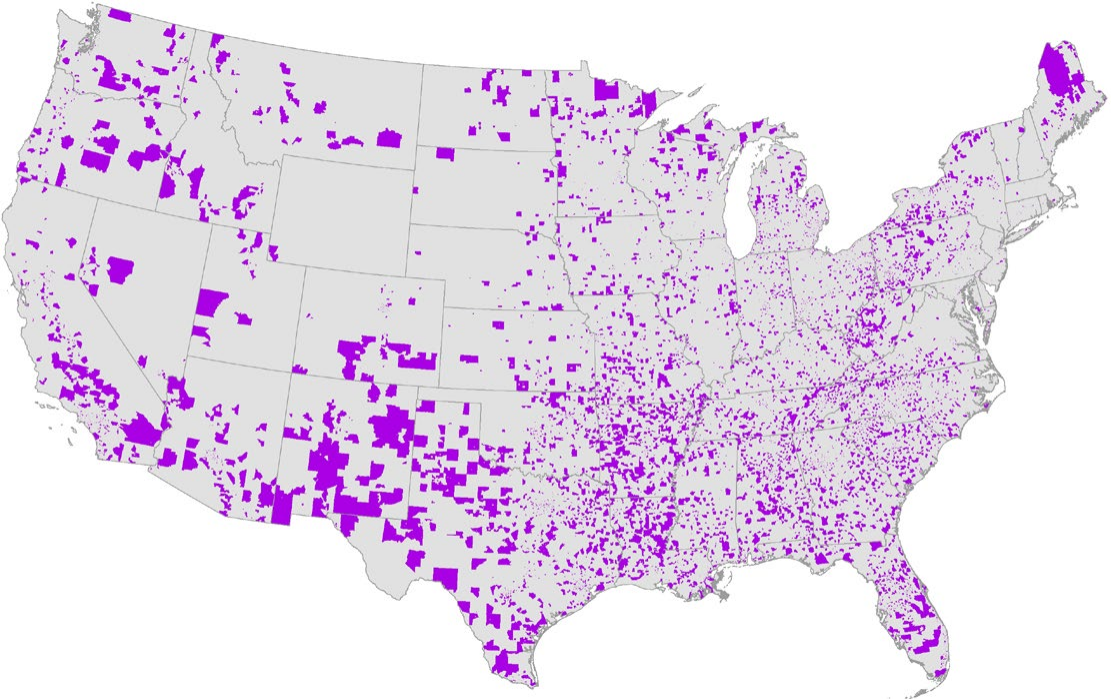
Map of the most disadvantaged neighborhoods of residence for medicare advantage enrollees eligible for control measures in 2013^†^ [Color figure can be viewed at http://www.wileyonlinelibrary.com/]
Notes. ^†^Neighborhood deprivation is derived from the Area Deprivation Index score, which was split into five equally sized quintiles. The most (5th) disadvantaged quintile is presented here. This map includes neighborhoods of residence for the entire study population of enrollees eligible for blood pressure, diabetes, and/or cholesterol control in 2013.

The fraction of plans’ enrollees who resided in the highest quintile of neighborhood disadvantage ranged from 0 to 63 percent for the blood pressure cohort (mean 16.3 percent; IQR: 6.1‐21.2 percent), 0‐70 percent for the diabetes cohort (mean 22.2 percent; IQR: 10.1‐30.9 percent), and 0‐69 percent for cholesterol cohort (mean 19.5 percent; IQR: 27.3‐75.6 percent). Enrollee neighborhoods of residence in the highest quintile of disadvantage were disproportionately located in the South and Midwest regions of the United States as well as in the most rural areas (Figure 1). For example, compared to the least disadvantaged neighborhoods, a higher proportion of enrollees in the most disadvantaged neighborhoods resided in the South (41 vs 6 percent; *P* < 0.001) or the Midwest (49 vs 8 percent; *P* < 0.001) in the diabetes cohort (Table 1).

**Figure 2:**
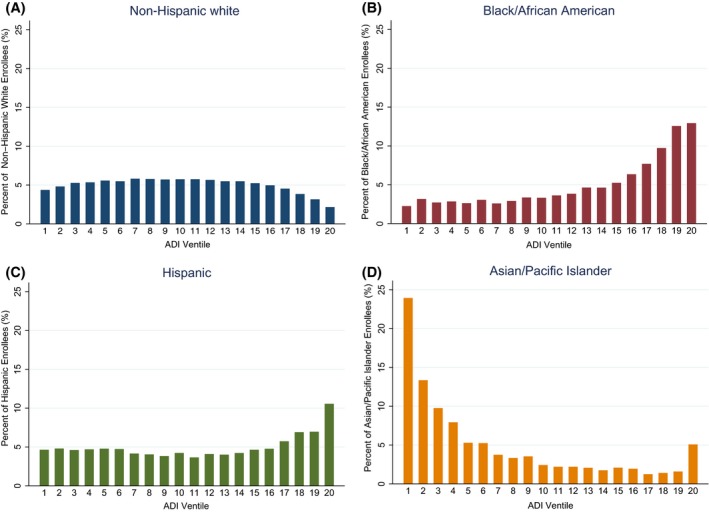
Number of white, black, hispanic, and asian enrollees in each ventile of neighborhood deprivation for blood pressure control^†^ [Color figure can be viewed at http://www.wileyonlinelibrary.com/]
Notes. ^†^Neighborhood deprivation is derived from the Area Deprivation Index score, which was split into 20 equally sized ventiles, representing increasing (worsening) neighborhood deprivation in 5 percent increments. Results not shown for diabetes and cholesterol control cohorts.

Across all three outcomes, the number of black enrollees in each ventile increased with worsening neighborhood disadvantage (Results for the blood pressure cohort shown in Figure 2). Conversely, the number of white enrollees in each ventile declined with worsening neighborhood disadvantage. These trends were most pronounced in the three most disadvantaged ventiles, or the top 15 percent most disadvantaged neighborhoods (Figure 2). The number of Asian enrollees decreased across ventiles, with a slight increase in the most disadvantaged ventile. The number of Hispanic enrollees increased with increasing neighborhood disadvantage, although this trend was less linear (Figure 2).

In unadjusted analyses, enrollees in the most disadvantaged neighborhood ventile were 10.3 percentage points (*P* < 0.05) less likely to have controlled blood pressure, 19.0 percentage points (*P* < 0.05) less likely to have controlled diabetes, and 26.4 percentage points (*P* < 0.05) less likely to have controlled cholesterol, compared with enrollees in the least disadvantaged ventile. Adjustment for individual and area‐level factors substantially attenuated, but did not eliminate, this relationship. After adjustment, enrollees in the most disadvantaged neighborhood ventile were 5.0 percentage points (*P* < 0.05) less likely to have controlled blood pressure, 6.9 percentage points (*P* < 0.05) less likely to have controlled diabetes, and 9.9 percentage points (*P* < 0.05) less likely to have controlled cholesterol, compared with enrollees in the least disadvantaged ventile (Table 2). Lastly, compared to the most urban areas, enrollees from the most rural areas were 11.8 percentage points (*P* < 0.05) less likely to have controlled blood pressure, 7.9 percentage points (*P* < 0.05) less likely to have controlled diabetes, and 8.6 percentage points (*P* < 0.05) less likely to have controlled cholesterol in adjusted models (Table 2). A sensitivity analysis revealed no difference in the results of adjusted models when enrollees missing an ADI score were excluded (Appendix S2: Table S2).

**Table 2:** Unadjusted and adjusted results: linear regression and generalized estimating equations^a^

Characteristics	Blood pressure control		Diabetes control		Cholesterol control	
	Unadjusted difference, % points (n = 182 012)	Adjusted difference, % points (n = 181 950)	Unadjusted difference, % points (n = 272 033)	Adjusted difference, % points (n = 271 944)	Unadjusted difference, % points (CI) (n = 200 356)	Adjusted difference, % points (n = 200 282)
Neighborhood deprivation						
Group 1 (least disadvantaged)	Reference	Reference	Reference	Reference	Reference	Reference
Group 2	−0.5	−0.0	−1.5*	−0.2	−3.3*	−1.5*
Group 3	0.4	0.8	−2.7*	−0.8	−4.8*	−1.2
Group 4	0.3	0.7	−2.9*	−0.8	−7.0*	−2.8*
Group 5	0.2	1.0	−3.6*	−1.2	−8.1*	−3.1*
Group 6	−1.0	0.1	−4.5*	−1.4	−10.3*	−4.3*
Group 7	−1.3	−0.0	−5.1*	−1.3	−11.1*	−4.2*
Group 8	−0.9	0.6	−6.5*	−1.9*	−13.6*	−5.7*
Group 9	−1.8*	0.1	−6.8*	−2.1*	−14.1*	−5.8*
Group 10	−2.4*	−0.3	−8.8*	−2.8*	−14.6*	−4.9*
Group 11	−2.0*	0.5	−8.5*	−1.8*	−16.4*	−5.7*
Group 12	−2.7*	0.2	−9.2*	−2.0*	−17.0*	−5.7*
Group 13	−4.9*	−1.4	−10.0*	−2.1*	−18.9*	−7.0*
Group 14	−5.7*	−1.8	−11.6*	−3.0*	−19.4*	−6.5*
Group 15	−5.2*	−0.9	−12.6*	−3.1*	−21.6*	−8.0*
Group 16	−6.5*	−1.5	−13.4*	−3.4*	−21.5*	−7.4*
Group 17	−7.5*	−1.5	−15.0*	−4.0*	−23.5*	−8.5*
Group 18	−9.0*	−2.5	−17.6*	−5.7*	−24.5*	−8.3*
Group 19	−11.3*	−4.3*	−17.7*	−5.1*	−25.8*	−8.6*
Group 20 (most disadvantaged)	−10.3*	−5.0*	−19.0*	−6.9*	−26.4*	−9.9*
Group 21 (missing Area Deprivation Index)	−5.2*	−1.0	−11.6*	−3.6*	−19.6*	−8.1*
Region						
Northeast	1.2*	1.4	−8.7*	−6.3*	−12.1*	−9.2*
Midwest	3.1 *	4.9*	−8.1 *	−4.6*	−14.1*	−9.9*
South	−8.7*	−5.9*	−13.9*	−9.7*	−19.7*	−14.8*
West	Reference	Reference	Reference	Reference	Reference	Reference
Sex						
Male	Reference	Reference	Reference	Reference	Reference	Reference
Female	−2.3*	−1.8*	0.1*	0.8*	−10.4*	−8.4*
Race/Ethnicity						
Non‐Hispanic white	Reference	Reference	Reference	Reference	Reference	Reference
Black/African American	−12.0*	−10.0*	−7.0*	−3.0*	−10.7*	−3.5*
Hispanic	−1.3*	−0.6	0.5*	−0.6	−1.5*	7.3
Asian/Pacific Islander	1.4*	−0.08	5.7*	0.5	10.1*	2.8*
American Indian/Alaska Native	−9.9*	−7.9*	−12.6*	−8.8*	−7.1*	−4.0
Other	−1.2	−2.4	5.6*	0.8	9.3*	3.2*
Unknown	−2.0	−3.7*	0.7	−1.1	4.6*	0.2
Dually enrolled						
Dual	−3.9*	−2.4*	−11.8*	−7.8*	−12.2*	−6.7*
Not dual	Reference	Reference	Reference	Reference	Reference	Reference
Original reason for Medicare						
Old age	Reference	Reference	Reference	Reference	Reference	Reference
Disability and/or ESRD	−2.1*	0.7	−12.1*	−7.8*	−10.1*	−5.5*
Rurality						
Large central metro (most urban)	−2.4*	−0.5	2.3*	0.7	1.7*	−0.2
Large fringe metro	Reference	Reference	Reference	Reference	Reference	Reference
Medium metro	−2.4*	−1.9	−3.4*	−2.8*	−2.0*	−1.6
Small metro	−7.9*	−7.7*	−5.1*	−4.6*	−5.8*	−4.5*
Micropolitan	−5.2*	−5.5*	−6.7*	−5.4*	−11.1*	−8.0*
Noncore (most rural)	−12.6*	−11.8*	−11.3*	−7.9*	−14.1*	−8.6*

a Unadjusted results are percentage point differences derived from bivariate linear regression coefficients. Adjusted results are percentage point differences derived from generalized estimating equation coefficients. All listed variables were included in the adjustment.

* *P* < 0.05.

**Figure 3:**
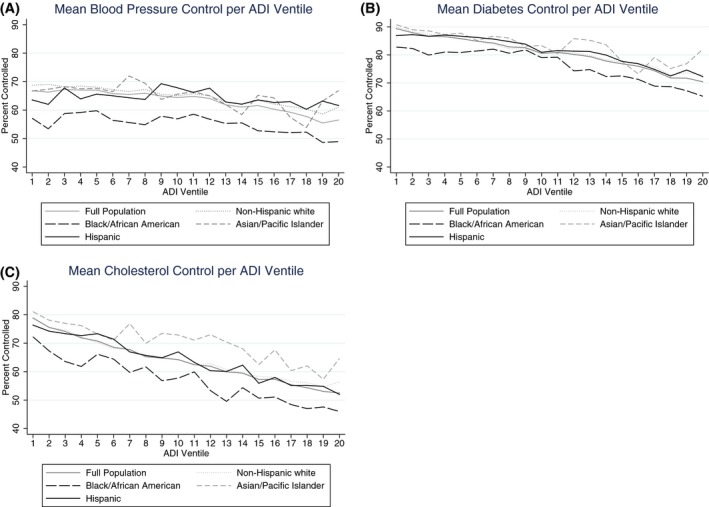
Proportion of enrollees achieving control of blood pressure, diabetes, and cholesterol with increasing neighborhood deprivation^†^ [Color figure can be viewed at http://www.wileyonlinelibrary.com/]
Notes. ^†^Neighborhood deprivation is derived from the Area Deprivation Index score, which was split into 20 equally sized ventiles, representing increasing (worsening) neighborhood deprivation in 5 percent increments. Each point plots the mean outcome control for either the entire population, or one racial/ethnic group in the population, over equally sized Area Deprivation Index ventiles that were created from the full population.

Although mean blood pressure, diabetes, and cholesterol control was lower for black enrollees than for other racial groups in every ventile, the overall trend between worsening outcome control and increasing neighborhood disadvantage did not differ by race (Figure 3). This finding was confirmed with a non‐significant interaction term for race and ADI score. However, we did find significant interactions between ADI score and dual eligibility as well as between ADI score and disability.

## 4 DISCUSSION

We found that that the ADI is a predictor of blood pressure, diabetes, and cholesterol control in the MA population. This relationship was attenuated, but persisted, after adjusting for area‐level and individual factors. The relationship between neighborhood disadvantage and blood pressure, diabetes, or cholesterol control did not differ by race or ethnicity.

Attenuation of neighborhood disadvantage in adjusted models may be due in part to the significant interaction between social risk factors other than race/ethnicity, including dual eligibility and disability. However, although the relationship between neighborhood disadvantage and blood pressure, diabetes, and cholesterol control was attenuated by adjustment for personal risk, there remained a small but significant correlation. This finding agrees with prior research that has found a relationship between neighborhood disadvantage and health outcomes after adjustment for individual social risk.^11^, ^14^

The relationship between neighborhood disadvantage and achievement of control varied across the three outcomes. The difference in outcome control between the most (ventiles 16‐20) and least (ventiles 1‐5) disadvantaged quintiles of neighborhood disadvantage was largest for the cholesterol and diabetes cohorts, and smaller in the blood pressure cohort. Only the top 10 percent (ventiles 19 and 20) most disadvantaged neighborhoods were significantly associated with worse blood pressure control after adjustment, indicating that this relationship is different for the most disadvantaged neighborhoods compared to the rest of the population (Table 2). This finding is consistent with a similar study of 30 day rehospitalizations in the Medicare population that identified worsening outcomes in the top 15 percent most disadvantaged neighborhoods.^10^ This difference may be explained in part by differences in cardiac risk factors between the blood pressure cohort and the cholesterol control cohort. Enrollees in the cholesterol control cohort must have a prior‐year history of a cardiac event, but eligibility for the blood pressure control measure only requires a diagnosis of hypertension. The authors of the 30‐day rehospitalization study hypothesized that at a certain threshold (the top 15 percent most disadvantaged neighborhoods), individuals who could previously compensate for neighborhood disadvantage could no longer do so, leading to worsening outcomes beyond this threshold.^10^ It is possible that the same is true for blood pressure control. Further research should explore the factors attenuating the relationship between blood pressure control and neighborhood disadvantage.

We identified disproportionate numbers of enrollees living in disadvantaged neighborhoods both in specific geographic regions, including the most rural areas, and concentrated within plans. We found that MA enrollees living in disadvantaged neighborhoods were more likely to be residents of rural rather than urban areas, and that these rural enrollees had worse control of blood pressure, diabetes, and cholesterol. This contrasts with the FFS population in which beneficiaries living within the most disadvantaged neighborhoods represent a mix of both rural and urban areas. This finding may be reflective of regional differences between MA and FFS enrollment patterns across the United States. However, our findings are consistent with prior research that demonstrates substantially higher risk factors for and mortality from cardiovascular disease among rural residents, particularly minorities. Such rural‐urban health disparities may be attributed in part to poor access to care and greater adverse burdens of social determinants of health in some rural areas.^21^ These findings are consistent with an accepted, multidimensional health disparities framework.^22^

In addition, we found a disproportionate number of black enrollees in the most disadvantaged neighborhoods. Although the segregation of black Americans in disadvantaged areas has been extensively described,^23^, ^24^ our work extends this literature to the MA population. However, the relationship between ADI and our health outcomes did not vary by race. For example, with each incremental decline in neighborhood disadvantage, the gap in health outcomes between black and white Americans remains stable. Yet, black Americans consistently have the lowest rates of blood pressure, diabetes, and cholesterol control across all ventiles, and are disproportionately represented in the most disadvantaged neighborhoods. This finding implies that interventions that are targeted to improve disadvantaged neighborhoods have the potential to improve health for all racial groups, but may not eliminate racial/ethnic health disparities unless they are focused on neighborhoods with higher fractions of minority residents.

Our findings agree with multiple prior studies that have found an independent relationship between neighborhood disadvantage and various health outcomes. A recent study using the ADI measure indicated that individuals in more disadvantaged neighborhoods have a 70 percent greater chance of hospital readmission than those in less disadvantaged neighborhoods even after adjustment for individual factors.^9^ Another recent study using a separate comprehensive index for neighborhood disadvantage found that heart failure is associated with worsening neighborhood disadvantage, also after adjusting for individual SES.^25^ Previous work also found that neighborhood disadvantage and coronary events remained associated after accounting for individual SES, although the relationship was slightly attenuated by the adjustment.^15^ In comparison with prior studies, we incorporated a more comprehensive measure of neighborhood disadvantage and also adjusted for measures of area‐level demographics, including rurality and geographic region. Further, our study extends this body of research to the MA population and to quality measures that are widely reported for most US insurers.

The incorporation of metrics of the social determinants of health into quality rankings is increasingly important to MA as such factors begin to be included in adjusted plan performance scores.^26^ This study identifies a social determinant of health measure that is publicly available, varies greatly across plans and regions, and is strongly associated with important quality outcomes that have been consistently collected and reported by MA plans. The ADI could also be used to target interventions at the most disadvantaged neighborhoods where MA enrollees live. This is particularly true for blood pressure control, where only the top 10 percent most disadvantaged neighborhoods were associated with the outcome measure. However, targeted interventions are less clear for diabetes and cholesterol control, which both have a linear relationship with neighborhood disadvantage. Addressing disparities in these outcomes may be better suited to risk adjustment strategies that take into account the proportion of enrollees in disadvantaged neighborhoods on a continuum.^27^ Given that MA plan performance on intermediate outcome measures is tied to financial benefits and penalties,^28^ investing in the improvement of highly disadvantaged neighborhoods may serve to both improve quality outcomes and financially benefit plans.

Several limitations should be noted. First, social determinants of health are a multidimensional construct, and the ADI does not include all relevant factors related to the social determinants of health. Second, Medicare collects imperfect data on socioeconomic indicators.^29^ Two Medicare indicators in this study, disability and dual eligibility, are proxies for disabled and low‐income groups, and may not completely capture the disadvantaged population. The disability measure may leave out enrollees who are disabled but are not enrolled in Social Security Disability Insurance, which is required for Medicare enrollment younger than 65. Similarly, the dual eligibility measure may leave out enrollees who are just below the threshold for dual or low‐income subsidy or Medicaid eligibility, or who were eligible but did not enroll in these programs.^29^ Third, this study does not answer the question of why worsening neighborhood disadvantage is associated with poor outcome control*,* but it is an important first step toward informing new interventions to address these outcomes. The association between neighborhood disadvantage and outcome control suggests that the social determinants of health included in the ADI are important contributors to poor health outcomes in disadvantaged areas. This finding could both inform further research on the mechanisms underlying this relationship and help target public health interventions at populations living in disadvantaged areas. Furthermore, using a composite measure of neighborhood disadvantage prevents the determination of which individual factors included in the index most contribute to poor outcome control in disadvantaged areas. Yet given the multidimensional causes of health disparities, it is likely that no one factor drives health outcomes equally in all areas of the United States.^22^ A composite measure may thus add value when studying a national sample. Fourth, this retrospective design evaluates current area of residence and cannot account for the effects of cumulative exposure to disadvantaged neighborhoods, which may account in part for health outcomes.^14^, ^15^, ^30^ Fifth, our findings may not generalize to the Medicare FFS program.

In conclusion, the ADI is a strong predictor of diabetes and cholesterol control, and a moderate predictor of blood pressure control. The ADI varies greatly across MA plans, and could potentially be used to track neighborhood‐level disparities and to target disparities‐focused interventions in the MA population.

## CONFLICT OF INTEREST

None.
