# Supplementary material for: Neighborhood disadvantage and chronic disease management
Source: Health Serv Res. 2018 Nov 23;54(Suppl 1):206–16. doi: 10.1111/1475-6773.13092 (PMC6341202; doi:10.1111/1475-6773.13092)
Supplement: Supplementary file 2 [file HESR-54-206-s002.docx]

**Appendix S1. Census Variables Included in the Area Deprivation Index**^†^

| Census Variable | Factor Score Coefficient* |
| --- | --- |
| Percent of the population aged 25 and older with less than 9 years of education | 0.0849 |
| Percent of the population aged 25 and older with at least a high school diploma | -0.0970 |
| Percent employed persons aged 16 and older in white-collar occupations | -0.0874 |
| Median family income in US dollars | -0.0977 |
| Income disparity | 0.0936 |
| Median home value in US dollars | -0.0688 |
| Median gross rent in US dollars | -0.0781 |
| Median monthly mortgage in US dollars | -0.0770 |
| Percent of owner-occupied housing units | -0.0615 |
| Percent of civilian labor force population aged 16 years and older who are unemployed | 0.0806 |
| Percent of families below federal poverty level | 0.0977 |
| Percent of the population below 150% of the federal poverty threshold | 0.1037 |
| Percent of single-parent households with children less than 18 years of age | 0.0719 |
| Percent of households without a motor vehicle | 0.0694 |
| Percent of households without a telephone | 0.0877 |
| Percent of occupied housing units without complete plumbing | 0.0510 |
| Percent of households with more than 1 person per room | 0.0556 |

^†^ The following individual variables are included in the composite Area Deprivation Index, as originally constructed by Singh using factor analysis.^1^

*Factor score coefficients represent the weight of the variable in the Area Deprivation Index. Poverty, income, and education have the largest weights.

**Appendix S2. Construction of the Study Population**

The Medicare Healthcare Effectiveness Data and Information Set (HEDIS) included 623,363 MA enrollees in 522 contracts in 2013 who were eligible for one or more dichotomous measures of blood pressure control, diabetes control, and/or cholesterol control. We matched 99% of these data to the Medicare Beneficiary Summary File (MBSF) (n = 622,652). From this initial data, we excluded enrollees with nine-digit ZIP Codes of residence outside of the United States (n = 15,713) and enrollees who died during the year of data collection (n = 1,400). Over 99.9% of the remaining data (n = 605,308) were matched to the 2013 NCHS Urban-Rural Classification Scheme for Counties. Lastly, eighty-five percent of these data were matched (n = 512,278) by nine-digit ZIP Code of residence to the Area Deprivation Index (ADI). The final dataset included 175,229 enrollees in 457 plans eligible for blood pressure control, 269,789 enrollees in 453 plans eligible for diabetes control, and 196,765 enrollees in 379 plans eligible for cholesterol control. There is some overlap in these populations as enrollees were eligible for one or more outcomes.

**Appendix S2: Table S1. Socioeconomic and Demographic Characteristics of Outcome-Eligible Medicare Advantage Enrollees, Missing and Non-Missing ADI Scores^†^**

|  | **Blood Pressure Control** | | **Diabetes Control** | | **Cholesterol Control** | |
| --- | --- | --- | --- | --- | --- | --- |
|  | **Not Missing ADI (n = 152,447)** | **Missing ADI (n = 29,565)** | **Not Missing ADI (n = 231,091)** | **Missing ADI (n = 40,942)** | **Not Missing ADI (n = 169,633)** | **Missing ADI (n = 30,723)** |
| Sex |  |  |  |  |  |  |
| Female | 57 | 55 | 51 | 49 | 37 | 35 |
| Race/Ethnicity |  |  |  |  |  |  |
| Non-Hispanic White | 67 | 71 | 57 | 62 | 70 | 74 |
| Black/ African American | 16 | 13 | 15 | 13 | 10 | 9 |
| Hispanic | 12 | 10 | 20 | 16 | 13 | 11 |
| Asian/Pacific Islander | 4 | 4 | 6 | 6 | 5 | 4 |
| American Indian/Alaska Native | 0 | 1 | 0 | 1 | 0 | 0 |
| Other | 1 | 1 | 2 | 2 | 1 | 1 |
| Unknown | 0 | 1 | 1 | 1 | 0 | 1 |
| Dual Eligibility |  |  |  |  |  |  |
| Dual Eligible | 27 | 25 | 23.9* | 23.7* | 19.1 | 19.7 |
| Original Reason for Medicare Enrollment |  |  |  |  |  |  |
| Disability and/or ESRD | 26 | 28 | 34 | 37 | 32 | 36 |
| Rurality |  |  |  |  |  |  |
| Most Urban (Large Central Metro) | 33 | 22 | 47 | 33 | 40 | 26 |
| Large Fringe Metro | 19 | 15 | 18 | 17 | 22 | 19 |
| Medium Metro | 27 | 24 | 21 | 21 | 24 | 23 |
| Small Metro | 10 | 14 | 7 | 11 | 7 | 11 |
| Micropolitan | 7 | 14 | 5 | 11 | 5 | 12 |
| Most Rural | 4 | 11 | 2 | 7 | 3 | 9 |
| Geographic Region |  |  |  |  |  |  |
| Northeast | 19 | 17 | 13 | 13 | 14 | 14 |
| Midwest | 21 | 19 | 14 | 14 | 15 | 15 |
| South | 34 | 34 | 23 | 25 | 29 | 33 |
| West | 26 | 30 | 50 | 48 | 41 | 38 |

^†^ All values are percentages and may not add to 100 due to rounding. Neighborhood deprivation is derived from the ADI score, which was split into twenty equally sized ventiles, with a separate category coded for enrollees missing an ADI score. The “Not Missing ADI” group includes all enrollees who were matched to an ADI score in any ADI ventile. All missing/not missing comparisons are significant at a level of p<.01, except where noted.

*p = 0.2

**Appendix S2: Table S2. Adjusted Results, With and Without Imputation^†^**

| **Characteristics** | **Blood Pressure Control** | | **Diabetes Control** | | **Cholesterol Control** | |
| --- | --- | --- | --- | --- | --- | --- |
|  | **Without Imputation (n = 152,396)** | **With Imputation (n = 181,950)** | **Without Imputation (n =231,022)** | **With Imputation (n =**  **271,944)** | **Without Imputation (n = 169,569)** | **With Imputation (n = 200,282)** |
| Neighborhood Deprivation | -2.1* | -4.9* | -1.2* | -3.5* | -3.7* | -3.8* |
| Region | -6.1* | -1.4* | 11.0* | 12.1* | 34.0* | 13.1* |
| Sex | 9.1* | 1.9* | -3.2* | -7.1* | 34.1* | 37.4* |
| Race/Ethnicity | -1.1* | -0.3* | -1.7* | -0.6* | 0.8* | 0.4 |
| Dually Enrolled | -12.5* | -2.8* | -46.3* | -43.4* | -22.1* | -25.4* |
| Original Reason for Medicare | -0.6 | -0.2* | -49.4* | -49.7* | -24.7* | -25.6* |
| Rurality | -5.8* | -1.4* | -13.2* | -10.7* | -8.4* | -7.1* |

**^†^** Adjusted results are derived from multivariable logistic regression, both with and without multiple imputation. Neighborhood deprivation was the imputed variable in the model. Neighborhood deprivation is derived from the ADI score, which was split into twenty equally sized ventiles, with a separate category coded for enrollees missing an ADI score.

*p<.05
